# Tactile stimulations and wheel rotation responses: toward augmented lane departure warning systems

**DOI:** 10.3389/fpsyg.2014.01045

**Published:** 2014-10-01

**Authors:** Christophe Tandonnet, Borís Burle, Franck Vidal, Thierry Hasbroucq

**Affiliations:** ^1^Faculté de Psychologie et des Sciences de l'Education, Université de GenèveGenève, Switzerland; ^2^Laboratoire de Neurosciences Cognitives, UMR 7291, CNRS, FR 3C FR 3512, Aix Marseille UniversitéMarseille, France

**Keywords:** driving, tactile reaction time, stimulus-response compatibility, practice, categorization

## Abstract

When an on-board system detects a drift of a vehicle to the left or to the right, in what way should the information be delivered to the driver? Car manufacturers have so far neglected relevant results from Experimental Psychology and Cognitive Neuroscience. Here we show that this situation possibly led to the sub-optimal design of a lane departure warning system (AFIL, PSA Peugeot Citroën) implemented in commercially available automobile vehicles. Twenty participants performed a two-choice reaction time task in which they were to respond by clockwise or counter-clockwise wheel-rotations to tactile stimulations of their left or right wrist. They performed poorer when responding counter-clockwise to the right vibration and clockwise to the left vibration (incompatible mapping) than when responding according to the reverse (compatible) mapping. This suggests that AFIL implements the worse (incompatible) mapping for the operators. This effect depended on initial practice with the interface. The present research illustrates how basic approaches in Cognitive Science may benefit to Human Factors Engineering and ultimately improve man-machine interfaces and show how initial learning can affect interference effects.

## Introduction

Thirty seven percent of all transportation fatalities in the USA are caused by running off from the road (National Highway Traffic Safety Administration: http://www.nhtsa.gov/NCSA). To cope with this problem, different major car companies (i.e.; Toyota, Honda, Audi, General Motors, Kia Motors, Nissan, Mercedes-Benz, BMW, Opel, PSA Peugeot Citroën) have developed “lane departure warning systems,” that are mechanisms designed to warn the driver when the vehicle is leaving its lane on freeways and arterial roads. The first function of such systems is to allow the driver to engage correcting movements on the steering wheel.

During the past two decades, basic research in Experimental Psychology and Cognitive Neuroscience has tremendously improved our knowledge of the brain mechanisms involved in action control. These advances are in the public domain and could be used to improve man-machine interactions. However, before practical recommendations can be formulated, predictions derived from theoretical constructs must be submitted to empirical testing. The present paper illustrates the first step of a research process that may ultimately augment lane departure warning system in automobile vehicles.

Stimulus-response compatibility (SRC) is a key factor for designing man-machine interfaces. It refers to the fact that some actions are easier or more difficult than others either because of the particular sets of stimuli and responses that are used or because of the way in which individual stimuli and responses are paired with each other (Kornblum et al., 1990). Hommel et al. proposed a general frame, the theory of event coding (TEC), that explains how stimuli and responses are represented and how these representations interact to generate SRC (Hommel et al., 2001; Hommel, 2009). At the core of TEC is the notion that produced actions (responses) are represented in terms of their perceptual consequences (stimulus). Responses and stimuli thus share some features in a common representational domain. Representations consist in composite feature codes organized in networks. The more features are shared by stimulus and response representations, the more compatible are the events they refer to. Feature overlap between stimuli and responses representations thus cause SRC effects.

Here, we shall focus to the lane departure warning system implemented in PSA Peugeot Citroën vehicles (AFIL, for “*alerte de franchissement involontaire de ligne*”) and bring empirical evidence that this system is potentially sub-optimal. In this system, the warning consists in a vibration delivered to the driver through the seat on the side of the lane departure. When receiving this warning, the driver must rotate the steering wheel so as to replace the vehicle in its lane (for studies on drivers' steering reactions to disturbances, see Muto and Wierwille, 1982; Wierwille et al., 1983; Franck et al., 1988). A first caveat of AFIL is to rely exclusively on the tactile modality. Multisensory displays that are based on the latest cognitive neuroscience research findings can capture driver attention significantly more effective than their unimodal (i.e., tactile) counterparts (for a review, see Spence and Ho, 2008). The second, and most critical point, is that AFIL delivers the tactile warning to the driver on the same side as the lane departure: when the vehicle runs off the lane on the left side, the left side of the drivers' seat is vibrated while when the vehicle runs off the lane on the right side, the right side of the drivers' seat is vibrated. Since different studies (Rieger et al., 2005; Sutter, 2007; Müsseler et al., 2008) showed that anticipated effects in external space are prevalent for SRC when using tools, delivering the warning on the side of the body corresponding to the lane departure (proximal reference) rather on the side of the to-be-performed corrective movement (distal reference) seemed a questionable option (for a similar analysis, see Straughn et al., 2009). It must further be stressed that “motor priming,” a recent prototype of device assistance, implements a mapping opposite to that of AFIL: In case of lane departure it delivers small alternating movements to the steering wheel directed toward the road center (Navarro et al., 2007, 2010).

The aim of the present study was to help deciphering the optimal way of delivering the tactile warning to the driver after the on-board system has detected a drift of the vehicle to the left or to the right. To this end, two stimulus-response mappings were contrasted. As AFIL, the first one favored the proximal references: It consisted in responding by a clockwise rotation of the steering wheel to the left-side stimulus and by a counter-clockwise rotation to the right-side stimulus. The second one favored anticipated effects in external space: It consisted in responding by a counter-clockwise rotation of the steering wheel to the left-side stimulus and by a clockwise rotation to the right-side stimulus.

According to TEC, the larger feature overlap between the stimulus and response representations, the larger the difference in performance between the mappings (Hommel, 2009). In AFIL, the lateralized warnings are delivered through the seat which does not insure maximal feature overlap between the stimulus and response representations. One way to augment this overlap so as to maximize the difference in performance between the two possible mappings consists in delivering the tactile warnings to a part of the body's driver directly involved in the steering rotation response. Here, in order to render the hypotheses testable with a limited number of participants, we chose to deliver the warnings to the wrists. This option was further technically easy to implement in a first approach. Future developments could be based on stimulating the palm of the hand through vibrators inserted in the steering wheel.

In driving conditions, any drift of the vehicle is inevitably accompanied with changes in the visual scene. In an attempt to design a situation closer to driving conditions, visual feed-backs relative to the orientation of the steering wheel were provided in the present study. The wheel movements therefore produced visual spatial effects entailing the confounding of stimulus-response compatibility with response-visual effect (feed-back) compatibility, which influence has been demonstrated (Hommel, 1993, 1996; Kunde, 2001; Kunde et al., 2002). For instance, Kunde (2001) demonstrated that keypresses are initiated faster when they trigger visual events in spatially corresponding rather non-corresponding locations. For the present purpose, the covariation of these two variables, which occurs under natural driving conditions, is unproblematic inasmuch it allows one to address the question of how the tactile information should be delivered to the driver and renders the experimental design more realistic.

While the results obtained in an applied study are unconclusive (Beruscha et al., 2010), basic research results (Guiard, 1983; Stins and Michaels, 1997; Proctor et al., 2004; Murchison and Proctor, 2013), lead us to expect the participant's performance to be better when the subjects responded counter-clockwise to left stimulations and clockwise to right stimulations than when they performed the reverse mapping. Compatibility being to a great extent a matter of learning (Kornblum et al., 1990), a second aim of the present study was to investigate how practicing the alternative mappings may affect the participants' performance.

## Materials and methods

### Participants

Twenty right-handed participants, 8 women and 12 men, aged 21–62 years (mean: 37, *SD*: 11), and holding a car driving license on average for 17 years (*SD*: 11, range 1–43) volunteered for the experiment. All of them had a normal or corrected-to-normal visual acuity. They were split into two groups of 10, each group comprised 4 women and 6 men. The participants of group 1 were aged 21–61 years (mean: 38, *SD*: 11) and were holding their car driving license on average for 18 years (*SD*: 12, range 1–43). The participants of group 2 were aged 25–62 years (mean: 36, *SD*: 12) and were holding their car driving license on average for 16 years (*SD*: 12, range: 3–42).

### Task

#### Apparatus and display

The participant was seated comfortably on a chair and was to grip with the two hands a Microsoft® Side winder® steering wheel, 26 cm in diameter, interfaced to a Pentium 4 equipped microcomputer. In front of the participant, behind the steering wheel, a 21 inches computer screen was disposed at eye level. This screen served to display visual feed-backs. The distance between the screen and the participants' eyes was about 70 cm. The stimuli were vibrations (Frequency 108 Hz, Amplitude 0.46 mm, duration 200 ms) applied to the internal part of the two wrists by electrical rotary engines (Deltron Euroind Company, Italy) inserted in cloth braces maintained by Velcro fixations. Responses were clockwise or counter-clockwise rotations of the steering wheel. Visual feedbacks were delivered on the computer screen. They consisted in triangles (Base = 9 cm, Height = 9 cm) of green (RGB = 19, 225, 0), blue (RGB = 0, 0, 255) or red (RGB = 255, 0, 0) color, carrying information detailed below.

#### Trial

Participants gripped the steering wheel with their hands in a typical driving position: their left and right hands being respectively on the left and right side of the wheel (10:10 grip). Each trial began by positioning the steering wheel in the starting position (between −64° and +0.64° with regard to the vertical). Once this position was reached, a green triangle pointing upward was displayed on the screen.

Provided that the steering wheel was kept in the starting position during 500 ms, a tactile stimulation was delivered either to the left or to the right wrist. The time allowed for the participants to leave the starting position and reach the target position was 800 ms. The target position was reached by rotating the steering wheel, either clockwise or counter-clockwise, depending on the stimulation (see below), to reach a position of 37.2° + 6.4° with regard to the vertical. The rotation of the steering wheel turned-off the green triangle and a blue triangle pointing either to the right, for a clockwise rotation, or to the left, for a counter-clockwise rotation, appeared on the screen. When the steering wheel reached the target position, the blue triangle turned green. If the steering wheel went too far (beyond the target), the green triangle turned red. All triangles were pointing to the right for a clockwise rotation and to the left for a counter-clockwise rotation. Participants had to maintain the steering wheel in the target position during 1000 ms. When they succeeded, the response was correct and an auditory positive feedback was emitted (Windows_XP_Sound_by_default.wav); otherwise, the response was incorrect and an auditory negative feedback was delivered (Windows_XP_ Discharged_battery.wav). A response was considered as an error when: (1) participants did not react in time (2) participants did not reach the target in time (3) participants reached the target but did not keep the position (4) participants went in the opposite direction.

### Instructions and mappings

Instructions were given verbally by the experimenter and emphasized both speed and accuracy. For one mapping, participants were asked to respond by a counter-clockwise rotation to the vibration of the left wrist and by a clockwise rotation to the vibration of the right wrist; for the other mapping, participants were asked to respond by a clockwise rotation to the vibration of their left wrist and by a counter-clockwise rotation to the vibration of their right wrist. Note that these formulations make no verbal reference to a possible lateral coding of the wheel-rotation responses.

### Design

The participants participated in four daily sessions. During each session, they performed first 15 warm-up trials and then 5 experimental blocks of 64 trials. The warm-up trials during each session were performed using the same mapping as the following five experimental blocks. Within a block, the two stimuli were equiprobable and delivered according to a pseudo-random sequence. The participants were given a few minutes of rest between each block. Mapping was alternated every other session. Group 1 began by responding counter-clockwise to the left stimulation and clockwise to the right stimulation; Group 2 did the reverse assignment of mapping to session (see Table 1).

**Table 1:** **Assignment of mappings to sessions for the two participants' groups**.

	**Group 1**	**Group 2**
Session 1	Left S / counter-clockwise R Right S / clockwise R	Left S / clockwise R Right S / counter-clockwise R
Session 2	Left S / clockwise R Right S / counter clockwise R	Left S / counter-clockwise R Right S / clockwise R
Session 3	Left S / counter-clockwise R Right S / clockwise R	Left S / clockwise R Right S / counter-clockwise R
Session 4	Left S / clockwise R Right S / counter clockwise R	Left S / counter-clockwise R Right S / clockwise R

*S, stimulus; R, response*.

### Data analysis

Mean RT was submitted to univariate repeated-measures analysis of variance (ANOVA). The design involved one between-subject factor, group (i.e., mapping sequence, two levels), and two within-subject factors, block of trials (five levels) and session (four levels). Mean error rates of “Side errors” (participants moved the steering wheel in the opposite direction) were arcsine transformed and submitted to analyses of variance with the same design as that used for the RT data. Other incorrect trials included “Late movements” (participants did not reach the target in time: with a movement time between 800 and 1600 ms), and “Stabilization” (participants reach the target but did not keep the position). These trials were also arcsine transformed and submitted to analyses of variance with the same design. Note that percentage data cannot be tested by parametric tests as their means and variances are closely related. However, the arcsine transform is efficient in stabilizing the variances of these data (Winer, 1970). In a few trials (<1%), participants did not react during the 800 ms following the stimulation; these omissions were judged too few for analysis.

## Results

### Reaction time

**Figure 1:**
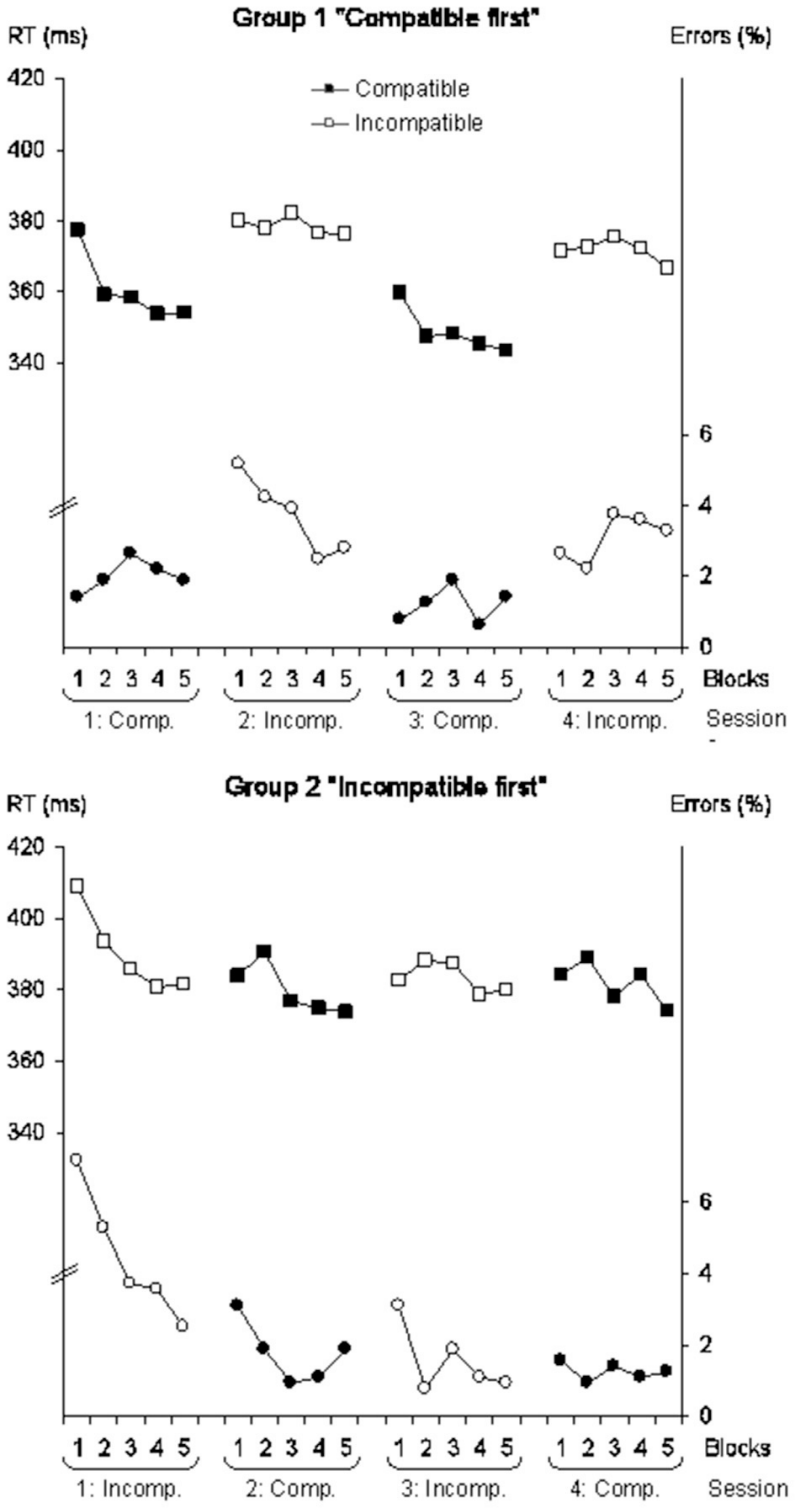
**Mean reaction time (in ms, upper part of each graphic, squares) and mean side error percentage (lower part, circles) for the group 1 practicing the compatible mapping first (“Compatible first,” top) and the group 2 practicing the incompatible mapping first (“Incompatible first,” bottom) as a function of block of trials, session, and mapping (compatible mapping: Comp., filled symbols; incompatible mapping: Incomp., empty symbols)**.

Mean RTs are presented in Figure 1 and Table 2. The participants of group 1 “Compatible first” responded by a counter-clockwise rotation to the left vibration and by a clockwise rotation to the right vibration during the first and third sessions while this mapping was performed by the participants of group 2 “Incompatible first” during the second and fourth sessions. Symmetrically, the participants of group 2 “Incompatible first” responded by a clockwise rotation to the left vibration during the first and third sessions while this mapping was performed by the participants of group 1 “Compatible first” during the second and fourth sessions. To test the effect of S-R mapping, we thus compared the first and third sessions together against the second and fourth sessions. This comparison revealed that the effect of session differed as a function of the group of participants [*F*_(1, 18)_ = 9.43, *p* = 0.01, η^2^ = 0.01].

**Table 2:** **Mean reaction time and percentage of incorrect trials sorted by type (Side errors, Late, Stabilization, Omission) for each group (Group 1 “Compatible first,” Group 2 “Incompatible first”) and for each condition (session, block of trials)**.

**Session (S)**	**Block**	**Group 1 “Compatible first” (G1)**						**Group 2 “Incompatible first” (G2)**					
		**RT (*M; SD*)**		**Side**	**Late**	**Stab**.	**Omis**.	**RT (*M; SD*)**		**Side**	**Late**	**Stab**.	**Omis**.
	1	378	39	1.4	2.5	7.0	0.0	409	78	7.2	1.9	7.3	0.6
S1	2	359	39	1.9	1.2	7.5	0.3	394	72	5.3	4.8	6.9	0.6
G1 Compatible	3	359	41	2.7	1.2	6.2	0.0	386	64	3.8	1.6	3.8	0.2
G2 Incompatible	4	354	42	2.2	0.6	6.1	0.2	381	58	3.6	2.7	4.5	0.0
	5	354	39	1.9	1.9	5.9	0.0	382	62	2.5	2.3	3.9	1.2
Mean		361	39	2.1	1.5	6.5	0.0	390	66	7.2	1.9	7.3	0.6
	1	380	56	5.2	1.7	5.9	0.5	384	67	3.1	0.6	3.0	0.8
S2	2	378	48	4.2	0.9	5.0	0.0	390	74	1.9	0.8	2.7	0.6
G1 Incompatible	3	382	49	3.9	1.4	3.4	0.3	377	62	0.9	1.1	1.2	0.2
G2 Compatible	4	377	55	2.5	0.8	4.4	0.2	375	67	1.1	1.2	0.9	0.5
	5	376	48	2.8	1.4	5.3	0.2	374	62	1.9	0.5	2.5	0.3
Mean		379	50	5.2	1.7	5.9	0.5	380	66	3.1	0.6	3.0	0.8
	1	360	26	0.8	0.2	4.8	0.0	383	66	3.1	1.1	2.2	0.0
S3	2	348	30	1.2	0.8	3.9	0.0	388	67	0.8	0.3	1.6	0.0
G1 Compatible	3	348	27	1.9	0.3	3.6	0.2	387	64	1.9	0.6	1.1	0.3
G2 Incompatible	4	346	29	0.6	0.5	3.4	0.0	379	62	1.1	0.5	1.7	0.8
	5	343	32	1.4	0.5	3.3	2.0	380	58	0.9	0.3	1.6	0.2
Mean		349	28	0.8	0.2	4.8	0.0	383	63	3.1	1.1	2.2	0.0
	1	372	36	2.7	0.3	4.5	0.0	384	63	1.6	0.5	2.2	0.8
S4	2	373	36	2.2	0.0	3.1	0.0	389	71	0.9	0.5	2.0	1.1
G1 Incompatible	3	375	38	3.8	0.3	5.2	0.0	378	64	1.4	0.2	0.5	0.2
G2 Compatible	4	372	35	3.6	0.9	4.8	0.2	384	67	1.1	0.3	2.7	0.9
	5	367	35	3.3	1.1	5.3	0.0	374	63	1.2	0.6	0.8	0.2
Mean		372	35	2.7	0.3	4.5	0.0	382	65	1.6	0.5	2.2	0.8
Mean (S1, S3)		355	31	1.4	0.8	5.7	0.0	387	64	5.2	1.5	4.8	0.3
Mean (S2, S4)		375	41	4.0	1.0	5.2	0.3	381	65	2.4	0.6	2.6	0.8

Conducted separately for each group, this comparison revealed that the participants of group 1 “Compatible first” reacted faster when they responded by a counter-clockwise rotation to left stimulation and a clockwise rotation to right stimulation than when they performed the reverse mapping [*F*_(1, 9)_ = 7.98, *p* = 0.02, η^2^ = 0.02]. Therefore, the former mapping was more compatible than the later one. Further comparisons allowed one to refine this analysis. For group 1 “Compatible first,” there was no significant difference in RT between the first and third sessions (compatible mapping) nor between the second and fourth sessions (incompatible mapping. *p*s > 0.10). In other words, the effect of S-R mapping was all-or-none: It was unaffected by the repetition of mapping sessions (see Figure 1).

Contrary to those of group 1 “Compatible first,” RTs of participants of group 2 “Incompatible first” who practiced the incompatible mapping during the first and third sessions were not significantly affected by the S-R mapping (*p* > 0.10). In addition, a direct comparison of the two groups revealed that mapping sequence did not significantly influence RT for the second and fourth sessions (*p* > 0.10), indicating that participants of group 2 “Incompatible first” in the compatible mapping reacted as slow as participants of group 1 “Compatible first” in the incompatible mapping. The two mappings appeared thus equally incompatible for the participants of group 2 “Incompatible first” (see Figure 1).

**Figure 2:**
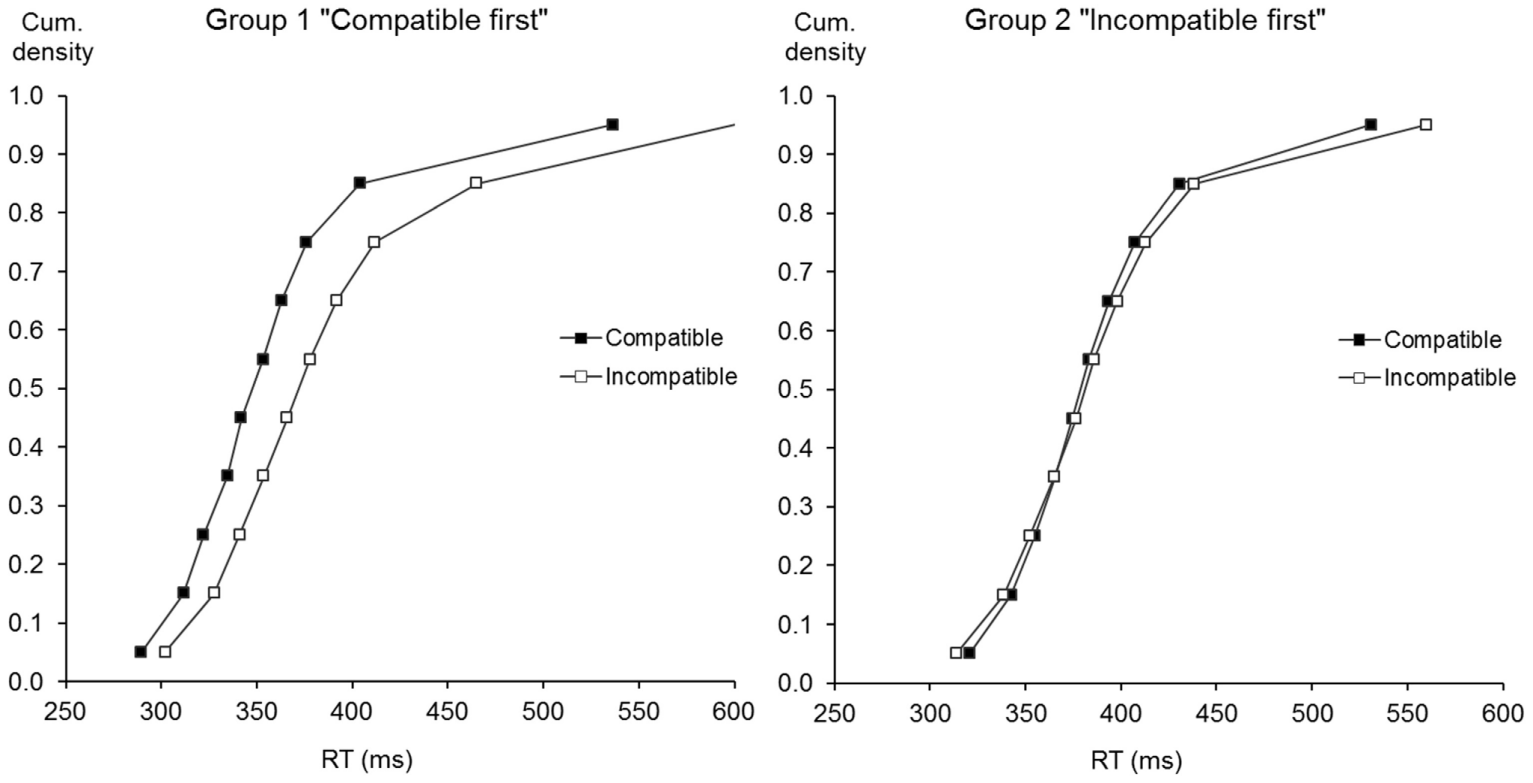
**Reaction time distribution**. Cumulative densities for the group 1 practicing the compatible mapping first **(top)** and the group 2 practicing the incompatible mapping first **(bottom)** for the last two experimental sessions as a function of mapping (compatible mapping: filled symbols; incompatible mapping: empty symbols).

In an attempt to better characterize the effects on mean RT, a distribution analysis was also performed. As the mapping effects can be partially confounded with those of with-session practice in the first two sessions, we focused the distributional analysis on the last two sessions. Individual RT distributions were “Vincentized” (Ratcliff, 1979); each individual RT distribution was binned in ten classes of equal size (same number of trials) and the mean of each bin was computed. Figure 2 presents the averaging of these individual RT distributions. It can be seen that participants of group 1 “Compatible first” consistently had a better performance for the compatible than for the incompatible mapping across all distribution deciles, contrary to the participants of group 2 “Incompatible first.”

### Error

#### Side errors

The side errors (participants moved the steering wheel in the opposite direction) represent 2.4% of the trials; mean side error percentages are presented in Figure 1. We compared the first and third sessions together against the second and fourth sessions for each group of participants, as for the reaction time. This comparison showed that the effect of session differed as a function of the group of participants [*F*_(1, 18)_ = 22.34, *p* < 0.01, η^2^ = 0.08]. This comparison conducted separately for each group revealed that, contrary to the participants of group 2 “Incompatible first,” the participants of group 1 “Compatible first” made significantly less errors when they were required to perform an counter-clockwise rotation when stimulated to the left wrist and a clockwise rotation when stimulated to the right wrist than when they were required to perform the reverse mapping [*F*_(1, 9)_ = 28.69, *p* < 0.01, η^2^ = 0.07; group 2: *F*_(1, 9)_ = 4.41, *p* = 0.07]. The former mapping was found more compatible than the latter one, which parallels the results on the reaction time (see Figure 1).

Further comparisons showed that, for group 1, there was no difference neither between the first and third sessions (compatible mapping) nor between the second and fourth sessions (incompatible mapping; *p*s > 0.10). For group 2, there was no difference between the second and fourth sessions (*p* > 0.10) but a significant difference between the first and third sessions [*F*_(1, 9)_ = 8.60, *p* = 0.02]. This pattern of results on side errors indicate an effect of S-R compatibility for the group 1 but an effect of between-session practice for the group 2 (see Figure 1).

#### Other incorrect trials

Late movements (participants did not reach the target in time: with a movement time between 800 and 1600 ms), Stabilization (participants reach the target but did not keep the position), and Omissions (participants did not react during the 800 ms following the stimulation) represent 3.8, 1.0, and 0.3% of the trials, respectively (Table 1). There were no significant effect of S-R mapping on “Late movements” and “Stabilization” trials, as assessed by the comparison between the first and third sessions together against the second and fourth sessions (*p*s > 0.10).

## Discussion

The participants of group 1, who initially responded counter-clockwise to the left vibration and clockwise to the right vibration, displayed a clear effect of compatibility on both RT and side error rate. This mapping remained more compatible than its alternative throughout the experiment. The participants of group 2, who first responded clockwise to the left vibration and counter-clockwise to the right vibration displayed no hint of compatibility effect. Thus, depending on initial practice, the mapping selected for AFIL (PSA Peugeot Citroën) was either neutral or detrimental to the participants' performance. A first implication of these findings is that it seems preferable to design systems requiring the operators to respond to left tactile stimulations by counter-clockwise wheel rotations and to right tactile stimulation by clockwise rotations. It can be stressed that this outcome is in line with predictions derived from TEC (Hommel, 2009) which posits that anticipated effects in external space (distal references) as opposed to body-centered representations (proximal references) are prevalent for SRC. One may object that the present result are not directly predictive of the drivers' behavior when using AFIL because, with this system, the lateralized tactile stimulations are delivered to the thighs rather than to the wrists. In a first approach, stimulating the wrists was intended to augment feature overlap between the stimuli and response sets in order to maximize the effect of mapping (Hommel, 2009) and allow conclusions from a limited sample of participants. The effect of this methodological choice should be directly addressed in forthcoming experiments, by delivering the warnings through the participants' seat. The hand position (crossed or uncrossed) could also play a role by modulating the compatibility between the tactile stimulation and the wheel-rotation responses. While hand placement plays a role for estimating the temporal order of successive tactile stimuli delivered at very brief intervals (Yamamoto and Kitazawa, 2001), previous research allows clear expectations relative to its influence on SRC. Since anticipated effects in external space are prevalent when using tools (Rieger et al., 2005; Sutter, 2007; Müsseler et al., 2008), best warning should probably be delivered in the direction of the to be performed rotation, irrespective of hand placement on the steering-wheel. The implications of the present results for designing on-board systems should further be confirmed in in more realistic driving conditions (in driving simulators and in real traffic circumstances). This may work when the driver hold a steering wheel with both hands in the horizontal position, i.e., the right hand in the position of 90-degree clockwise from the top and the left hand in the position of 90-degree counterclockwise. This might be a natural position of hands when holding the steering. But, we must notes that there are wide variation in the position of hand. When turning a left bent, the right hand can be in the top of wheel and the left hand in the bottom of wheel. Even, the right hand can be more left and the left hand can be more right when the driver crosses both hands.

Another important issue for future research is to combine the tactile stimulation with an information relative to the vehicle position that is often delivered in motorways: A rumble strip on the lane marker produces a vibration when the car is running off from the lane. The relationship between the tactile stimulus and this vibration can be formalized in terms of stimulus-stimulus compatibility (SSC, Fitts, 1954). Another instance of SSC likely interfered with SRC in an experiment conducted in a driving simulator by Straughn et al. (2009). These authors examined the effect of compatibility between steering responses and accessory left/right tactile signals delivered after visual imperative signals. In this task, the compatibility between the accessory and the response (SRC) and the compatibility between the imperative signal and the accessory (SSC) covaried in opposition. The delay between the imperative signal and the accessory signal was also manipulated. For short delays, the left accessory—counter clockwise response / right accessory clockwise response led to the best level of performance. In contrast, for long delays, the alternative mapping led to the best performance level. While disentangling the respective effects of the two compatibility relationships is beyond the scope of the present paper, it must be stressed that for the short delay condition that is the closest to the present experiment, the effect of accessory-response compatibility corresponds to the SRC effects evidenced in the present study. The inversion of this effect for long delays shows that the relationship between SRC and SSC should be addressed before firm conclusions relative to the use of onboard lane departure systems can be reached (for an application of this notion in ergonomics, see Akamatsu et al., 1995).

The efficiency of vibrations were compared to that of the “motor priming” device (Navarro et al., 2007) that prompts drivers to take action by means of small asymmetric oscillations (Navarro et al., 2010) producing either a clockwise or a counter-clockwise rotation of the steering wheel. Motor priming implements a compatible mapping: a clockwise rotation calls for a clockwise response and a counter-clockwise rotation counter-clockwise response (toward the road center). Surprisingly, the authors tested motor priming against left and right tactile vibrations incompatibly mapped with the steering response: the left vibration was to be responded to by a clockwise movement and the right vibration by a counter-clockwise movement (toward the lane departure). The present results suggest that motor priming would be better tested against a compatible vibrotactile-motor mapping.

A final comment relative to automotive ergonomics is in order. So far, lateral control assisting devices can be split in two categories: lane departure warning system and lane keeping assistance systems. Lane departure warning systems, such as that tested in the present study, simply inform the driver that the vehicle is in an unsafe lane position. Lane Keeping assistance systems actively intervene on the steering wheel. For instance, torque can be continuously applied to the steering wheel to help the driver to keep close to the center of the lane. Future developments based on motor priming concept (Navarro et al., 2007) may combine the two types of systems. This can be done by taking advantage of the torque already installed for power steering and may render obsolete systems based on auditory or vibratory warnings (for a recent review see Beruscha et al., 2011).

We suggest that instead of considering from the beginning complex near-to-real man-machine interactions, Human Engineering starts from facts from basic research even established in unpoverished experimental conditions. Applied research could then elaborate validation processes for testing the relevance of these facts under increasing complexity situations progressively approaching real-life conditions. This issue is of societal relevance for such validation processes may hopefully save human lives, in particular in transportations.

The list-rule model of SRC (Hasbroucq et al., 1990) is a functional model compatible with the representational component of TEC (Hommel, 2009). It provides a simple systematic articulation between stimulus-response match and the effect of mapping. Functionally, like other instances of SRC proper (e.g., Fitts and Deininger, 1954), the present effect can be attributed to a central response selection process which transforms the stimulus categorized by preceding identification processes into an abstract response code that is subsequently transmitted to motor stages of information processing (Proctor and Reeve, 1990), a notion that is supported by data from single cell recordings in behaving monkeys (Riehle et al., 1997; Mouret and Hasbroucq, 2000). According to the list-rule model, when the stimuli and responses sets do not match, they are differently categorized and the only response selection procedure available to the subject consists of scanning the memorized list of the individual stimulus-response pairs that have been defined in the instructions as correct. In this case, no effect of mapping is expected. If, alternatively, the stimulus and response sets match, the subjects do commonly categorize the stimuli and responses and the possibility of an algorithmically governed response selection can emerge. In particular, when the mapping associates the counter-clockwise response to the left stimulus and the clockwise response to the right stimulus, the common categorization of stimuli and response in lateral terms allows the subjects to select their responses by applying the identity transformation.

We shall now consider the implications of the effect of mapping sequence for this model and for man-machine interfaces. Indeed, only the participants of group 1 exhibited an effect of SRC. According to the list-rule model, these participants initially completed a mapping fitting their *a priori* left-right categorization of the stimulus and response sets. A difference in response selection procedure across sessions accounts for the emergence of an effect of SRC in this group. In sessions one and three, the subjects of group 1 applied the identity transformation while, in sessions two and four, this simple rule was no more available. They were thus to select their responses either by applying the inverted transformation to the stimuli categorized in left and right terms or to scan the memorized list of the alternative stimulus-response pairs: left / clockwise, right / counter-clockwise. In contrast, the subjects of group 2 exhibited no effect of compatibility. In terms of the list-rule model, these subjects initially completed a mapping that did not fit their *a priori* left-right categorization and they resorted to the same response selection procedure across experimental sessions. Admittedly, for the reason evoked above, in sessions one and three, the subjects of group 2 scanned the memorized list of the two alternative stimulus-response pairs. Surprisingly, their performance did not improve in sessions two and four during which the stimuli and responses could perfectly be categorized in lateral terms in order to apply the identity rule. The absence of performance improvement in these sessions suggests that the scanning procedure implemented in session one and three overrode the available common categorization of the stimulus and response sets, thereby preventing the use of the identity transformation which would have made emerged an SRC effect comparable to that observed for group 1.

These findings suggest that the categorization processes depend not only on the pre-experimental background of the subjects but also on the mapping according to which the experimental task is initially performed. Indeed, our results indicate that pre-experimental background and experimental practice interact, thereby determining the categorization operated by the subjects throughout the experiment. From a human factors perspective, an implication of this finding is that the first experience with an interface may be critical: it can determine the subsequent performance of individuals with this interface. Taking compatibility factors into account should thus help designing the most favorable learning procedure when operators are confronted with new man-machine interfaces. Since industrial and transportation contexts incur serious potential hazards, this may have strong societal implications. Since optimal man-machine interactions are conditioned by the compatibility of the mapping initially prescribed on a given interface, operators should learn the most compatible mapping. To this aim, the relative compatibility of all possible mappings should be assessed so as learning procedures favor the most compatible one. Finally, in complex situations, determining which associations will be congruent and which ones will not cannot rely on naïve judgments (Payne, 1995; Vu and Proctor, 2003; Hoffmann, 2010) but must be evaluated experimentally. It is noteworthy that such evaluations must take into account learning effects.

With respect to SRC research, there is not much evidence in the literature that learning has pronounced effects on SRC (e.g., Brebner, 1973; Dutta and Proctor, 1992) and the present effect of practice is not easy to reconcile with the functional distinction between long-term and short-term stimulus-response associations advocated by Barber and O'Leary (1997). Recent studies show, however, that preliminary practice of incompatible associations in tasks where the stimulus location is relevant does modify the effect of the irrelevant stimulus location in subsequent tasks (e.g., Tagliabue et al., 2000; Proctor et al., 2003). The present results demonstrate comparable transfer effects when the stimulus location of the subsequent task is relevant.

### Conflict of interest statement

The authors declare that the research was conducted in the absence of any commercial or financial relationships that could be construed as a potential conflict of interest.
